# Targeting G6PD with Benzimidazole and Thiazole Derivatives Suppresses *SIRT 2* and *VEGF* Expression and Induces Cytotoxicity in Glioma Cells

**DOI:** 10.3390/ijms26189092

**Published:** 2025-09-18

**Authors:** Montserrat Vázquez-Bautista, Laura Morales-Luna, Verónica Pérez de la Cruz, Rosa Angélica Castillo-Rodríguez, José Antonio Velázquez-Aragón, Sergio Enríquez-Flores, Luis Antonio Flores-López, Elizabeth Hernández-Urzúa, Víctor Martínez-Rosas, Carlos Wong-Baeza, Isabel Baeza-Ramírez, Gabriel Navarrete-Vázquez, Benjamin Pineda, Beatriz Hernández-Ochoa, Saúl Gómez-Manzo

**Affiliations:** 1 Laboratorio de Bioquímica Genética, Instituto Nacional de Pediatría, Secretaría de Salud, Mexico City 04530, Mexico; montsevazquez97@gmail.com (M.V.-B.);; 2 Programa de Posgrado en Biomedicina y Biotecnología Molecular, Escuela Nacional de Ciencias Biológicas, Instituto Politécnico Nacional, Mexico City 11340, Mexico; 3 Posgrado en Ciencias Biológicas, Universidad Nacional Autónoma de México, Mexico City 04510, Mexico; 4 Neurobiochemistry and Behavior Laboratory, National Institute of Neurology and Neurosurgery “Manuel Velasco Suárez”, Mexico City 14269, Mexico; veped@yahoo.com.mx; 5 Centro de Investigación en Ciencia Aplicada y Tecnología Avanzada (CICATA) Unidad Morelos, Instituto Politécnico Nacional, Boulevard de la Tecnología, 1036 Z-1, P 2/2, Atlacholoaya 62790, Mexico; 6 Laboratorio de Oncología Experimental, Instituto Nacional de Pediatría, Ciudad de Mexico 04530, Mexico; jovear2002@gmail.com; 7 Laboratorio de Biomoléculas y Salud Infantil, Instituto Nacional de Pediatría, Secretaría de Salud, Mexico City 04530, Mexico; sergioenriquez@ciencias.unam.mx; 8 Secretaría de Ciencia, Humanidades, Tecnología e Innovación (SECIHTI)-Instituto Nacional de Pediatría, Secretaría de Salud, Mexico City 04530, Mexico; luisbiolexp@gmail.com; 9 Laboratorio de Toxicología Genética, Instituto Nacional de Pediatría, Secretaría de Salud, Mexico City 04530, Mexico; elyzabet91@yahoo.com.mx; 10 Departamento de Ingeniería Química y Bioquímica, Instituto Tecnológico de Milpa Alta, Tecnológico Nacional de México, Mexico City 12300, Mexico; ing_vicmr@hotmail.com; 11 Laboratorio de Biomembranas, Departamento de Bioquímica, Escuela Nacional de Ciencias Biológicas, Instituto Politécnico Nacional, Mexico City 11350, Mexico; charlywong@icloud.com (C.W.-B.); isabelbaeza@yahoo.com (I.B.-R.); 12 Facultad de Farmacia, Universidad Autónoma del Estado de Morelos, Cuernavaca 62209, Mexico; gabriel_navarrete@uaem.mx; 13 Neuroimmunology Laboratory, National Institute of Neurology and Neurosurgery “Manuel Velasco Suárez”, Mexico City 14269, Mexico; benjamin.pineda@innn.edu.mx; 14 Laboratorio de Inmunoquímica, Hospital Infantil de México Federico Gómez, Secretaría de Salud, Mexico City 06720, Mexico

**Keywords:** glioblastoma, G6PD, hypoxia, cytotoxic compounds

## Abstract

Hypoxia and activation of the pentose phosphate pathway (PPP), as well as overexpression of glucose-6-phosphate dehydrogenase (G6PD), are hallmark features of glioblastomas (GBM), contributing significantly to tumor progression metabolic adaptation and drug resistance. This study aimed to evaluate the cytotoxic effects of nine synthetic compounds incorporating annulated benzimidazole and nitrothiazole scaffolds in two glioblastoma cell lines (A172 and U87-MG) under both normoxic and hypoxic conditions. Three compounds (BZM-7, BZM-9, and CNZ-3) demonstrated potent anticancer activity, with CNZ-3 exhibiting the highest efficacy, particularly in hypoxia. The study further investigated the effects of these compounds on the expression of the *G6PD* gene, as well as post-translational regulatory genes *SIRT2* and *KAT9*, and the angiogenesis-related *VEGF* gene. Transcriptional analyses showed that the nitrothiazole-derived compound CNZ-3 significantly downregulated *G6PD*, *SIRT2*, *KAT9* and *VEGF* expression under hypoxic conditions, suggesting selective interference with hypoxia-adaptative pathways. In contrast, BZM-7 and BZM-9 showed distinct expression patterns, indicating diverse mechanisms of action despite structural similarity. In addition, BZM-7, BZM-9, and CNZ-3 were identified as potent inhibitors of recombinant G6PD, demonstrating both enzymatic inhibition and structural alterations, suggesting that G6PD could be a relevant therapeutic target for these compounds. Furthermore, molecular docking analysis revealed favorable binding interactions between the compounds and key amino acids of the G6PD, reinforcing their potential as a direct enzyme inhibitors. These findings highlight the pivotal role of G6PD in gliomas under hypoxic conditions and support its inhibition as a promising therapeutic strategy.

## 1. Introduction

Glioblastoma multiforme (GBM) is the most common and aggressive malignant primary brain tumor in adults, arising from glial cells-particularly astrocytes. It is characterized by rapid proliferation, diffuse infiltration, and resistance to standard therapies [1,2,3,4]. These features contribute to its poor prognosis with median overall survival ranging from 14 to 18 months despite the multimodal treatment, including surgical resection followed by radiotherapy and chemotherapy with temozolomide (TMZ; an alkylating agent that induces both single- and double-strand DNA breaks) [5,6,7,8,9,10]. The cellular and molecular heterogeneity, as well as its ability to adapt metabolically to microenvironmental stressors, plays a key role in therapeutic resistance and recurrence [11].

Among the many factors that define tumor microenvironment, hypoxia plays a critical role in metabolic reprogramming. Hypoxic stress not only limits the effectiveness of therapies [12,13] but also promotes tumor progression through the activation of hypoxia-inducible factor 1-alpha (HIF-1α), a transcription factor that induces angiogenesis by upregulating vascular endothelial growth factor (VEGF) expression [14,15,16]. HIF-1α also drives the transcription of genes involved in glycolysis and glucose uptake, redirecting cellular metabolism toward lactate production, bypassing mitochondrial oxidative phosphorylation, thereby supporting tumor cells survival under low-oxygen conditions [17].

A recent study has highlighted the dynamic interplay between glycolysis and the pentose phosphate pathway (PPP) in glioma stem-like cells (GSCs), which can switch between these metabolic routes depending on oxygen availability [18]. Under normoxic conditions, these cells upregulated PPP, while hypoxia enhances glycolytic flux. A key regulator of the PPP is glucose-6-phosphate dehydrogenase (G6PD), which catalyzes the first and rate-limiting step pathway [19]. The *G6PD* gene is significantly overexpressed in a variety of tumor cells, including gliomas, and the activity of G6PD and expression is strongly correlated with tumor cell proliferation and poor patient outcomes [20,21,22,23,24,25,26]. These findings suggest that G6PD may serve as a potential diagnostic and therapeutic target in cancer.

Beyond transcriptional regulation, G6PD activity is also regulated by post-translational modifications (PTMs) such as phosphorylation, acetylation, glycosylation, ubiquitination, and glutarylation [27]. Among these, lysine acetylation has drawn particular interest due to its evolutionarily conserved role in regulating nuclear transcription and cytoplasmic metabolism [28,29,30]. The balance between acetylation by lysine acetyltransferase (KAT9, which inhibits G6PD activity), and deacetylation by SIRT2 (which reactivates G6PD) controls PPP flux and the production of cytosolic NADPH, which is essential for redox homeostasis and cancer cell survival under oxidative stress [31,32,33]. Despite extensive evidence showing upregulation of G6PD expression and activity across various tumors, its regulation through acetylation/deacetylation by KAT9 and SIRT2 has not yet been studied in hypoxic glioma cells. Moreover, the role of this regulatory mechanism in supporting glioma cell proliferation and redox homeostasis remains unknown.

Considering the pivotal role of G6PD in tumor metabolism and survival, we investigated the pharmacological effects of nine synthetic compounds, including seven benzimidazole-based derivatives, one nitrothiazole derivative, and a phenylpropanoic acid analog on glioblastoma cell lines (A172 and U87-MG) under both normoxic and hypoxic conditions. We examined their effects on the expression of *G6PD*, *SIRT2*, *KAT9*, and *VEGF*, key regulators of metabolism and angiogenesis. Finally, we evaluated the potential of G6PD as a therapeutic target of these compounds by inhibition assays, and through in silico studies, we made predictions of the binding of the compounds with the best antiproliferative activity (BZM-7, BZM-9 and CNZ-3).

## 2. Results and Discussion

### 2.1. Identification of Candidate Compounds with Cytotoxic Activity in A172 and U87-MG Cell Lines

**Figure 1:**
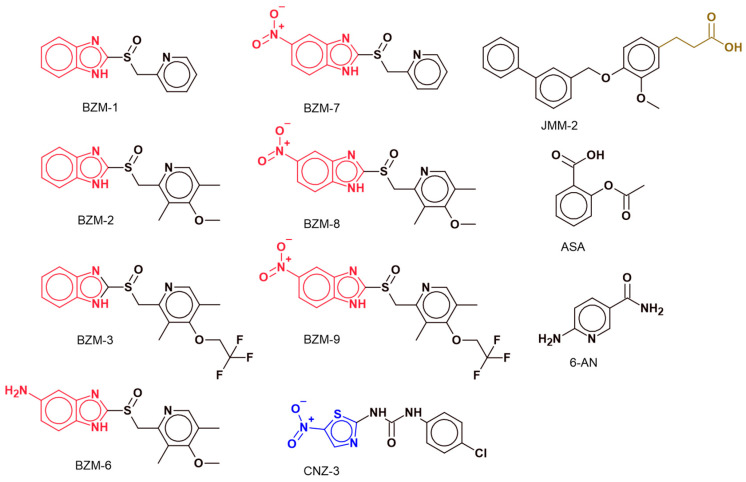
Chemical structures of the compounds. The benzimidazole ring, present in compounds BZM-1, BZM-2, BZM-3, BZM-6, BZM-7, BZM-8, and BZM-9, is highlighted in red, with each compound featuring variations in their chemical substituents. In contrast, CNZ-3 contains a thiazole ring, highlighted in blue. The structures of acetylsalicylic acid (ASA) and 6-aminonicotinamide (6-AN) are also included for comparison. All chemical structures were drawn using ACD/ChemSketch version 2020.2.0.

GBM remains one of the most aggressive and lethal forms of brain cancer, with limited effective therapeutic options and poor prognosis. In search of novel therapeutic agents, we evaluated the cytotoxic potential of a panel of a series of nine chemical compounds on two established human glioblastoma cell lines (A172 and U87-MG). The synthetic compounds included seven benzimidazole-based derivatives (BZM-1, BZM-2, BZM-3, BZM-6, BZM-7, BZM-8, and BZM-9), one nitro-thiazole derivative (CNZ-3), and a phenylpropanoic acid derivative containing a biphenyl scaffold (JMM-2). To provide comparative reference, we included 6-aminonicotinamide (6-AN), a nicotinamide analog and competitively inhibits G6PD, to suppress proliferation in various types of cancer cells [34,35]. Additionally, acetylsalicylic acid (ASA) was included due to its documented ability to acetylate multiple cellular proteins, promote apoptotic pathways [36,37,38,39,40,41,42], and inhibit angiogenesis [43,44]. The chemical structures of the compounds are shown in Figure 1.

CNZ-3 is a thiazole derivative, characterized by the presence of a thiazole ring substituted with a nitro group and a chlorophenyl ring, joined by a urea-type bond. In contrast, BZM-7 and BZM-9 are benzimidazole derivatives—heterocyclic compounds composed of a fused benzene and imidazole ring. The benzimidazole scaffold is known for its structural versatility and broad range of pharmacological applications. These compounds are considered pharmacologically active due to their ability to interact with diverse biological targets [45]. Notably, benzimidazole derivatives are currently under investigation as potential chemotherapeutic agents, particularly for the treatment of drug-resistant cancers such as breast cancer [46].

**Figure 2:**
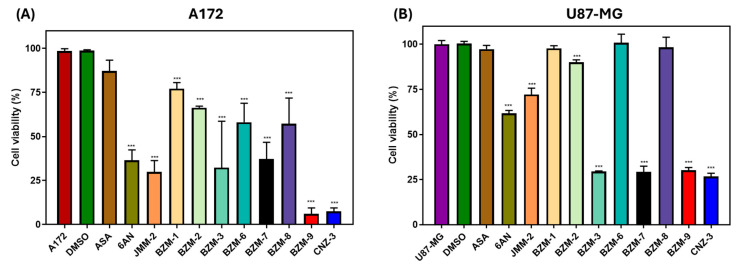
Effect of synthetic compounds on cell viability in glioblastoma cells. Cell viability of (**A**) A172 and (**B**) U87-MG glioblastoma cell lines after 48 h of treatment with synthetic compounds at a fixed concentration of 250 µM. Viability was assessed using the MTT assay. Data are expressed as the mean ± standard deviation from three independent experiments, and errors were less than 5%. Mann–Whitney U statistical test. *** *p* < 0.001 vs. untreated cells.

Since TMZ has been reported to exhibit IC_50_ values of 275.6 µM and 242 µM under normoxic and hypoxic conditions, respectively [47]. We performed an initial screening of compounds at a concentration of 250 μM to select those that induced a 50% reduction in cell viability. When cells were treated with 250 µM of each compound, a notable reduction in cell viability was observed in both A172 and U87-MG glioblastoma cell lines, with distinct compound-specific responses, shown in Figure 2. In the A172 cell line, BZM-9 and CNZ-3 showed the most pronounced cytotoxic effect, each reducing viability by 92%. Other compounds, including BZM-3, BZM-7, and JMM-2, also showed considerable cytotoxicity, reducing viability by 68%, 63%, and 70%, respectively. The known G6PD inhibitor 6-AN reduced viability by 64%, supporting the potential role of G6PD inhibition in the observed effects. In contrast, BZM-1, BZM-2, BZM-6, BZM-8, and ASA exhibited limited cytotoxicity, reducing viability by less than 50% (Figure 2A).

In the U87-MG cell line, CNZ-3 exhibited the highest cytotoxic activity, reducing cell viability by approximately 74%. BZM-3, BZM-7, and BZM-9 followed closely, each inducing around 70% reduction in viability. In contrast, 6-AN and JMM-2 demonstrated low cytotoxicity, reducing viability by 35% and 30%, respectively. BZM-1, BZM-2, BZM-6, BZM-8, ASA, did not show cytotoxic effect, and as expected the vehicle control DMSO did not affect the viability of the cells (Figure 2B).

These results identify BZM-7, BZM-9, and particularly CNZ-3 as consistently effective across both cell lines, each reducing cell viability by more than 50%. CNZ-3, demonstrating the strongest cytotoxic activity overall, highlights its potential as a promising therapeutic candidate. Based on these findings, BZM-7, BZM-9, and CNZ-3 were selected for further evaluation in subsequent experiments.

### 2.2. Determination of IC_50_ Values Under Normoxic and Hypoxic Conditions

**Figure 3:**
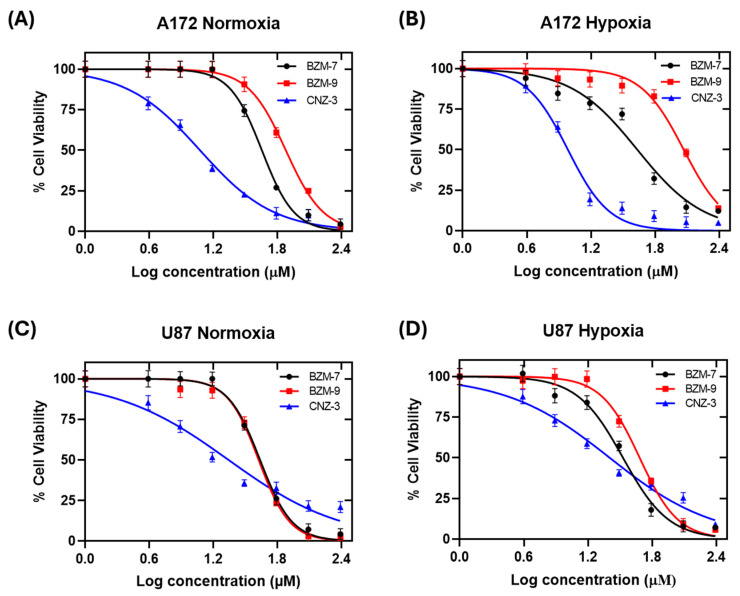
Concentration–response curves of BZM-7, BZM-9, and CNZ-3 in A172 and U87-MG glioblastoma cell lines under normoxic and hypoxic conditions. Cells were exposed to increasing concentrations of each compound for 48 h under normoxic (Panel (**A**): A172, Panel (**C**): U87-MG) and hypoxic (Panel (**B**): A172, Panel (**D**): U87-MG) conditions. Cell viability was assessed using MTT assay, and IC_50_ values were calculated.

GBM remains one of the most lethal brain tumors, largely due to its aggressive nature, high recurrence rate and strong resistance to therapy [48]. A hallmark of GBM is the presence of hypoxia within the tumor microenvironment, which plays a critical role in tumor progression, therapy resistance and metabolic reprogramming [49,50,51,52]. To further assess the antitumor potential of the three most promising compounds identified in our previous screening—BZM-7, BZM-9, and CNZ-3, we determined their inhibitory concentration 50 (IC_50_) in A172 and U87-MG glioblastoma cell lines under both normoxic and hypoxic conditions. As shown in Figure 3, all three compounds demonstrated a concentration-dependent decrease in cell viability.

As shown in Table 1, CNZ-3 emerged as the most potent compound in A172 cells, showing the lowest IC_50_ values under both normoxic and hypoxic conditions. This highlights its strong and consistent cytotoxic activity, regardless of oxygen availability. In contrast, BZM-9 lost the efficacy under hypoxia, suggesting that its activity may be more sensitive to changes in oxygen availability. In the U87-MG cell line, CNZ-3 also maintained strong cytotoxic; however, a modest increase in IC_50_ value was observed under hypoxia, indicating a slight reduction in potency. BZM-7 maintained similar efficacy in both conditions, while BZM-9, as in A172 cells, showed a slight reduction in potency.

All three compounds outperformed the standard chemotherapeutic agent temozolomide (TMZ), whose IC_50_ value has been reported as 275.6 µM and 242 µM under normoxic and hypoxic conditions, respectively [47]. Among the tested compounds, CNZ-3 demonstrated around 23-fold-greater potency compared to TMZ (IC_50_ = 12 µM in A172 and U87-MG under normoxia). This was followed by BZM-7 (~7-fold) and BZM-9 (~5-fold). These findings underscore the strong cytotoxic activity of these compounds, particularly CNZ-3, even under hypoxic conditions that typically confer chemoresistance. The structural classification of these compounds provides further insight into their potential. BZM-7 and BZM-9 are nitrobenzimidazole derivatives, a class of compounds known for their broad anticancer activity. CNZ-3 is a nitrothiazole derivative, a scaffold previously associated with cytotoxicity and apoptosis induction in cancer models. Several studies have highlighted the ability of nitrobenzimidazole and nitrothiazole compounds to inhibit cancer cell proliferation and induce apoptosis, supporting their value as promising anticancer agents [53,54,55,56,57].

### 2.3. Gene Expression Analysis of SIRT2, KAT9, VEGF and G6PD in Glioblastoma Cells Under Normoxia and Hypoxia

GBM is characterized by pronounced metabolic reprogramming, often driven by hypoxic conditions within the tumor microenvironment. Among the key metabolic pathways affected, the PPP plays a crucial role in maintaining redox balance, regulating cell growth and death, and contributing to tumor progression [58]. G6PD, the rate-limiting enzyme of the PPP, has been found to be upregulated in various tumor types, including glioblastoma [59]. To investigate the transcriptional regulation of G6PD and its modulators, we evaluated the expression levels of *G6PD*, *SIRT2* (a deacetylase), *KAT9* (a lysine acetyltransferase), and *VEGF* (a canonical hypoxic-responsive gene) in A172 and U87-MG glioblastoma cell lines under normoxic and hypoxic conditions. The cell line HMC3, which lacks tumor characteristics, was used as a non-cancerous control.

**Figure 4:**
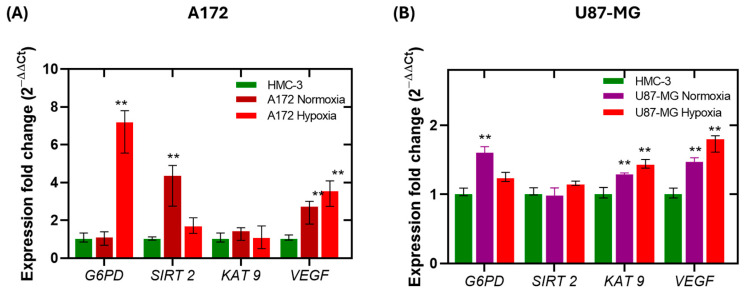
Relative mRNA expression levels of *G6PD*, *SIRT2*, *KAT9*, and *VEGF* in (**A**) A172 and (**B**) U87-MG glioblastoma cell lines under normoxic and hypoxic conditions. Gene expression was quantified by RT-qPCR and normalized to pyruvate kinase M1/2 (PKM) as the reference gene. Expression levels are presented relative to the non-cancerous HMC3 cell line. Data represents the mean ± standard deviation (SD) of three independent replicates. The double asterisk (**) indicates a significant difference (*p* < 0.05 vs. control normoxia or hypoxia) in expression based on the Mann–Whitney test.

As shown in Figure 4A, under normoxia, the expression levels of *G6PD* and *KAT9* in A172 cells were similar to those in HMC3. However, *SIRT2* and *VEGF* were upregulated by 3.3-fold and 1.7-fold, respectively. Under hypoxic conditions, *G6PD* expression increased by 6-fold, and *VEGF* by 2.5-fold, while *SIRT2* showed a moderated increases (0.6-fold) compared to control, and *KAT9* expression remained unchanged. These findings align with literature suggesting that hypoxia promotes the expression of *G6PD* and *VEGF* to support oxidative stress adaptation and angiogenesis [60,61,62,63,64]. For instance, overexpression of *SIRT2* has been shown to suppress *VEGF* expression in head and neck cancer cells, suggesting that *SIRT2* may negatively regulate hypoxia-induced *VEGF* expression [62]. This inverse relationship between *SIRT2* and *VEGF* expression under hypoxia may point to a compensatory regulatory mechanism. Furthermore, the hypoxia-induced elevation of *G6PD* suggests enhanced PPP to maintain NADPH production and cellular redox balance. Given that *SIRT2* activates G6PD by deacetylation and KAT9 inhibits it through acetylation, this expression dynamic suggest that the acetylation/deacetylation axis may be a key regulatory node in hypoxia-mediated metabolic reprogramming.

In contrast, U87-MG cells exhibited a more attenuated transcriptional response to hypoxia (Figure 4B). The expression of *G6PD*, *SIRT2*, and *KAT9* increased only slightly (0.2-fold, 0.1-fold, and 0.4-fold, respectively), and *VEGF* expression rose by ~0.8-fold under hypoxic conditions. These findings indicate that A172 cells are more transcriptionally responsive to hypoxic stress, particularly in the genes studied, whereas U87-MG cells may possess a lower sensitivity to oxygen deprivation or a more metabolic adaptability.

The overexpression of the *G6PD* gene in the cell lines evaluated is consistent with that found in clinical glioma samples, where *G6PD* expression is higher compared to that of normal cells. Furthermore, *G6PD* overexpression has been reported to be related to tumor grade, where *G6PD* expression levels were found to increase from 5- to 245-fold change in clinical samples grades 1 and 2, while in grade 3 and 4 samples, the increase was a 2197- to 4946-fold change [58]. This suggests that the PPP is an important pathway for glioma cells, as it provides the necessary precursors for nucleic acid synthesis in glioma cells, allowing rapid growth and proliferation, which are characteristic of grade 3 and 4 gliomas.

Together, these results suggest that *G6PD*, *SIRT2*, and *KAT9* play important roles in the hypoxic adaptation of glioblastoma cells. The stark contrast between A172 and U87-MG cell responses underlines the importance of considering inter-cell line variability in drug sensitivity and gene regulation. Based on these insights, we next investigated the effect of BZM-7, BZM-9, and CNZ-3 on the expression of *G6PD*, *SIRT2*, *KAT9*, and *VEGF* under both normoxic and hypoxic conditions, aiming to elucidate their potential impact on key metabolic and angiogenic pathways in GBM.

### 2.4. Differential Regulation of G6PD, SIRT-2, KAT 9, and VEGF by Test Compounds Under Normoxic and Hypoxic Conditions

**Figure 5:**
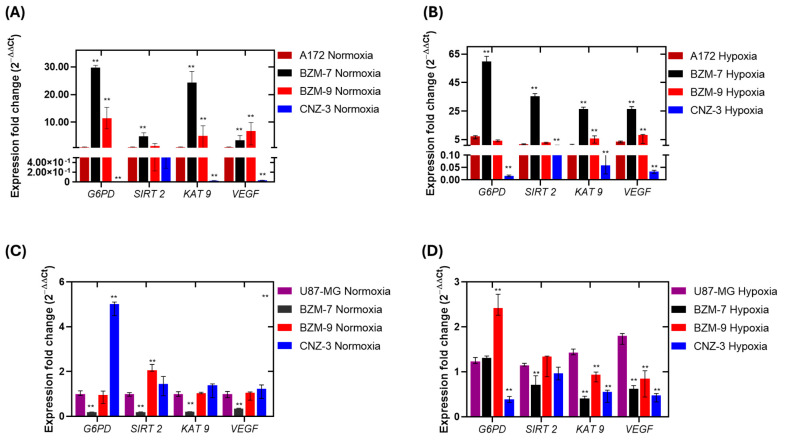
Relative expression of *G6PD*, *SIRT 2*, *KAT 9*, and *VEGF* in (**A**,**B**) A172; (**C**,**D**) U87-MG cell lines following treatment with BZM-7, BZM-9 and CNZ-3. Gene expression was assessed by RT-qPCR and compared to that of the HMC3 microglial cell line (non-cancerous control), and glioblastoma cells (negative controls). *Pyruvate kinase* was used as a reference gene. Data represents the mean ± SD of three replicates. The double asterisk (**) indicates a significant difference (*p* < 0.05 vs. control normoxia or hypoxia) in expression based on the Mann–Whitney test.

Gene expression analysis following treatment with the IC_50_ concentrations of BZM-7, BZM-9, and CNZ-3 under normoxic and hypoxic conditions revealed distinct transcriptional responses in both A172 and U87-MG glioblastoma cell lines (Figure 5). Overall, the benzimidazole derivatives BZM-7 and BZM-9 induced upregulation of *G6PD*, *SIRT2*, *KAT9*, and *VEGF* under both oxygen conditions. In contrast, the nitrothiazole derivate CNZ-3 consistently triggered a marked downregulation of these genes. These findings demonstrate that each compound induces a unique transcriptional profile response, modulated by the cellular oxygen status.

In A172 cells, BZM-7 treatment under normoxia led to upregulation of *G6PD* (28-fold), *SIRT2* (4-fold), *KAT9* (23-fold), and in *VEGF* (3-fold), relative to untreated cells. Under hypoxia, expression levels further increased for *G6PD*, *SIRT2*, and *KAT9* (58-, 34-, and 26-fold change, respectively), and *VEGF* (25-fold), indicating that BZM-7 activates pathways associated with redox regulation, metabolic adaptation and angiogenesis in both oxygen conditions. In contrast, for BZM-9, it elicited a more moderate transcriptional response. Under normoxia, *G6PD* expression increased by 11-fold; however, under hypoxia, it only rose 4-fold. Interestingly, *VEGF* under BZM-9 treatment showed no statistically significant difference between normoxic and hypoxic conditions Figure 5A,B. This attenuation may indicate that BZM-9 interferes with the typical hypoxia-induced transcriptional upregulation of genes involved in angiogenesis and metabolic. Although BZM-9 did induce *G6PD* expression, the reduced response under hypoxia—where G6PD is typically upregulated—suggests that the compound could disrupt the metabolic reprogramming usually triggered by low oxygen availability. By preventing the full activation of G6PD under hypoxia, BZM-9 could impair the PPP, which is essential for maintaining NADPH levels and counteracting oxidative stress. This disruption of redox homeostasis under stress conditions may contribute to the cytotoxic effect observed by this compound. Overall, these observations highlight the important mechanistic differences between BZM-7 and BZM-9, despite their structural similarity. While both compounds activate similar genes, BZM-7 consistently enhances their expression in both oxygen conditions environments, whereas BZM-9 showed variable, oxygen dependent effects, particularly on *G6PD* and *VEGF*.

The most striking effects were observed with CNZ-3, which exhibited the lowest IC_50_ values in both cell lines, consistent with its potent cytotoxic effect. In contrast to the benzimidazole derivatives, CNZ-3 markedly reduced the expression of *G6PD*, *KAT9*, and *VEGF* in both oxygen conditions. In A172 cells under normoxia, CNZ-3 downregulated *G6PD* (83-fold), *KAT9* (48-fold), and *VEGF* (26-fold), compared to untreated cells. Similar effects were seen under hypoxia (62-fold for *G6PD*, 17-fold for *KAT9,* and 30-fold for *VEGF*). While *SIRT2* expression was also reduced by CNZ-3, although the effect was more moderate (Figure 5A,B). These results strongly suggest that CNZ-3 interferes with hypoxia-inducible pathways and may impair angiogenic signaling and metabolic adaptation under low oxygen availability [65]. In particular, its potent suppression of *G6PD* under hypoxia implies a disruption of PPP activity, thereby compromising antioxidant defense mechanisms essential for glioma cell survival under stress conditions [66]. The oxygen-dependent transcriptional repression induced by CNZ-3 highlights its therapeutic potential as a targeted inhibitor of hypoxia-driven metabolic reprogramming in glioblastoma.

In the U87-MG cell line, treatment with BZM-7, BZM-9, and CNZ-3 compounds under normoxic and hypoxic conditions revealed a distinct pattern of gene expression regulation compared to the cell line A172, underscoring the importance of cellular context in drug response. BZM-7 treatment led to a downregulation of all four genes under both oxygen conditions, except for *G6PD*, whose expression remained unchanged under hypoxic conditions. These findings suggest that BZM-7 may be more effective in disrupting metabolic and stress response pathways under normoxia in U87-MG cells. This inverse expression pattern implies that BZM-7 may act through regulatory pathways that are more susceptible to suppression in U87 cells, which are known to be less hypoxia-responsive compared to A172.

Following BZM-9 treatment under normoxia, *G6PD* expression decreased ~1-fold, while *SIRT2* increased ~2-fold. *KAT9* and *VEGF* levels remained unchanged. Under hypoxia, however, *G6PD* and *SIRT2* levels increased by 2.4- and 1.3-fold, respectively, while *KAT9* and *VEGF* expressions decreased (1.8- and 2-fold, respectively). These results are particularly relevant considering that the IC_50_ for BZM-9 was higher in U87-MG (IC_50_ = 48 μM) than in A172 cells (IC_50_ = 35 μM), indicating lower cytotoxicity in this context. Nonetheless, BZM-9 still activated stress-related transcriptional programs. The observed induction of G6PD, a key enzyme in the PPP, under hypoxia may reflect an adaptive mechanism aimed at maintaining redox balance and NADPH homeostasis.

CNZ-3 exerted the strongest gene-suppressive effects. In hypoxic U87-MG cells, it significantly downregulated *G6PD*, *SIRT2*, *KAT9*, and *VEGF* by 3-, 1.8-, and 2.4- and 2.6-fold, respectively, relative to untreated controls. In contrast, under normoxia, CNZ-3 slightly increased the expression of *G6PD*, *KAT9*, and *VEGF* (5-, 1.3-, and 1.2-fold, respectively). *SIRT2* expression was upregulated ~1.4-fold in normoxia but remained unchanged under hypoxia. These data suggest that CNZ-3 modulates redox-related gene expression in an oxygen-dependent manner, particularly suppressing the antioxidant response and angiogenic signaling under hypoxic stress.

Taken together, these findings indicate that CNZ-3 exerts a potent gene-suppressive effect, in contrast to BZM-7 and BZM-9 and selectively impacts pathways critical for hypoxia adaptation. Its ability to downregulate *G6PD* and *VEGF*, particularly under hypoxic conditions, may contribute to its enhanced cytotoxicity by impairing cellular adaptation mechanisms such as redox balance and angiogenesis. This hypothesis aligns with previous findings by Kosuke Funato et al. 2018, who demonstrated that *SIRT2* knockdown in glioblastoma cells suppresses proliferation and tumorigenicity, induces cell cycle arrest, and promotes apoptosis, indicating that SIRT2 deacetylase activity is essential for GBM cell survival [67]. At the protein level, SIRT2 inhibitors have been shown to prevent the formation of active G6PD dimers [68], which decreases NADPH concentrations. Furthermore, inhibitors also promote the ubiquitination and degradation of c-Myc, and exhibit broad anticancer activity [69]. In turn, c-Myc is a key transcription factor in cell proliferation, activating *G6PD* gene expression, thereby increasing the activity of this enzyme and the flow of the PPP, as well as promoting cell cycle progression in cancer cell proliferation [70]. Therefore, compounds that show negative effects on *SIRT2* and *G6PD* expression could be promising candidates for further study as potential therapeutic agents.

Although BZM-7 and BZM-9, which feature benzimidazole rings, did not significantly suppress expression of the evaluated genes, they showed notable cytotoxic activity, suggesting a different mechanism of action compared to CNZ-3. The benzimidazole ring is an essential pharmacophore in various physiologically active heterocyclic compounds, since it has been reported to interact with various biological targets through metal ion interactions, π–π stacking and hydrogen bonding [71]. Therefore, benzimidazole derivatives have garnered significant interest as potential anticancer agents. The biological activity of benzimidazoles occurs through interaction with DNA, enzyme inhibition, and modulation of cellular pathways crucial for cancer development [72,73,74,75].

It is noteworthy that BZM-7, BZM-9, and CNZ-3 exhibited greater antiproliferative activity in GBM cells than the standard chemotherapeutic temozolomide (TMZ). Notably, BZM-9 and CNZ-3 suppressed *G6PD* expression under both oxygen conditions, while BZM-7 did so in normoxia. Furthermore, CNZ-3 had previously been reported as a non-competitive inhibitor for the G6PD enzyme, so this enzyme could be a potential target of this compound [76]. The next objective was to evaluate whether BZM-7, and BZM-9, were potent inhibitors of G6PD protein enzymatic activity.

### 2.5. Effect of Compounds on the Recombinant Glucose-6-Phosphate Dehydrogenase (G6PD)

#### 2.5.1. Determination of IC_50_ Values

**Figure 6:**
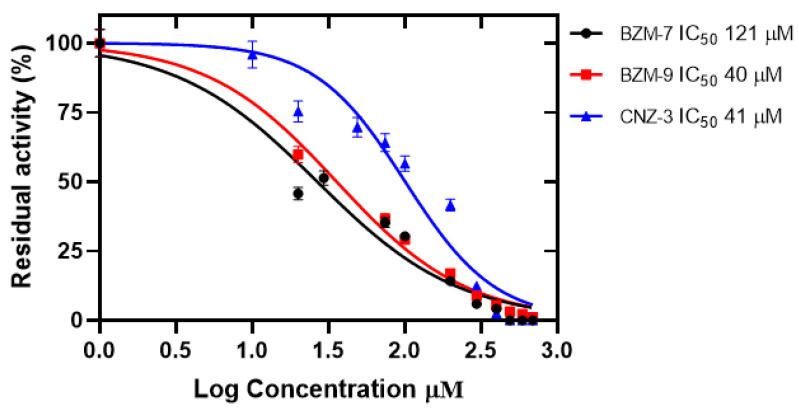
Inactivation of recombinant human Glucose-6-Phosphate Dehydrogenase (G6PD) by synthetic compounds. The compounds BZM-7, BZM-9, and CNZ-3 were analyzed. The recombinant enzyme was incubated at 0.2 mg/mL with increasing concentrations (0–800 µM) of each compound (BZM-7, BZM-9, and CNZ-3) for 2 h at 37 °C. IC_50_ values were determined by constructing concentration–response curves, plotting residual enzymatic activity against compound concentration. All experiments were performed in triplicate to ensure reproducibility of the results. Values represent the mean ± SD of three replicates.

Enzyme inhibition assays were conducted using recombinant human G6PD to evaluate the inhibitory potential of the compounds BZM-7, BZM-9, and CNZ-3. A concentration–response assay was performed to determine the concentration required to reduce G6PD enzymatic activity by 50% (IC_50_). As shown in Figure 6, all three compounds inhibited G6PD in a concentration-dependent manner. The calculated IC_50_ values were 40 µM for BZM-7, 42 µM for BZM-9, and 121 µM for CNZ-3, indicating that BZM-7 and BZM-9 were the most potent inhibitors, requiring the lowest concentration to achieve 50% inhibition of G6PD activity. These findings are consistent with the cellular expression assays, in which BZM-7 and BZM-9 significantly reduced G6PD transcript levels, particularly under normoxic conditions.

The results suggest that G6PD is a potential molecular target of these compounds. Notably, the IC_50_ values for all three compounds were considerably lower than those of previously reported G6PD inhibitors such as 1-dehydroepiandrosterone (DHEA), with an IC_50_ of 483 µM [77], as well as JMM-2 (307 µM), CCM-4 (412 µM), and CNZ-7 (274 µM) [76]. Given the critical role of G6PD in maintaining cellular redox homeostasis, biosynthesis, and chemoresistance in cancer cells, its inhibition represents a promising therapeutic strategy in glioblastoma [78,79]. G6PD overexpression contributes to tumor growth and survival, while its suppression can sensitize cancer cells to chemotherapy and radiotherapy [80,81]. Therefore, the ability of BZM-7, BZM-9, and CNZ-3 to inhibit G6PD enzymatic activity supports their potential as therapeutic candidates for therapy in glioblastomas.

However, it is also important to evaluate the effects of compounds on healthy cells, since inhibition of the enzyme glucose-6-phosphate dehydrogenase (G6PD) in non-cancerous cells—particularly in red blood cells—can induce oxidative stress and hemolysis, leading to hemolytic anemia, as observed in patients with G6PD deficiency [76]. This occurs because G6PD plays an essential role in the production of NADPH, a molecule that protects cells against oxidative damage. Without sufficient NADPH, cells become highly vulnerable to oxidative stressors, such as some medications, infections, or foods (e.g., fava beans), which can result in symptoms such as jaundice, fatigue, and shortness of breath [76]. Therefore, these effects should be evaluated in the future.

#### 2.5.2. Structural Alterations by Circular Dichroism

**Figure 7:**
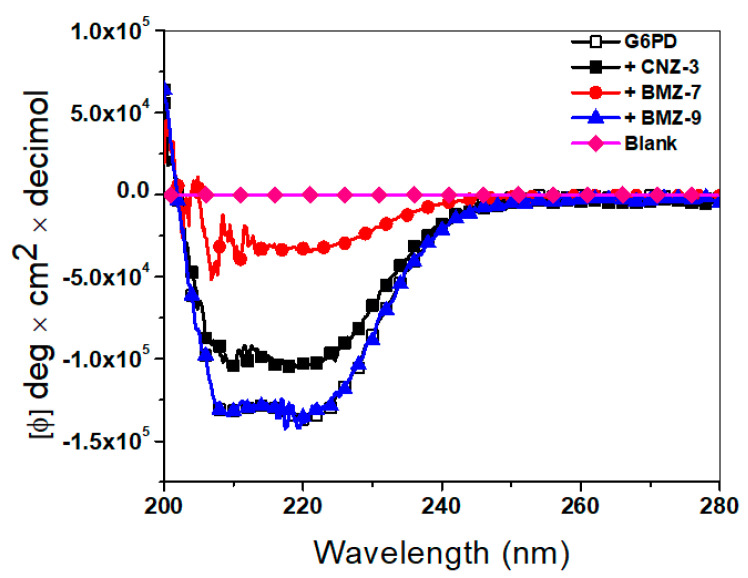
Circular dichroism (CD) spectra of recombinant human Glucose-6-Phosphate Dehydrogenase (G6PD). The enzyme was prepared at a concentration of 0.2 mg/mL in 50 mM phosphate buffer, pH 7.35, and incubated in the presence of the IC_50_ value determined for each compound. Structural characterization was performed in the far-UV region (200–260 nm). The results shown are representative of experiments performed in triplicate. Values represent the mean ± SD of three replicates.

Given that the synthetic compounds exhibited a negative effect on the catalytic activity of recombinant human G6PD, we investigated the structural alterations induced by these compounds at the secondary structure level using circular dichroism (CD) assays. As shown in Figure 7, the native enzyme (in the absence of compounds) exhibited typical CD spectra with negative ellipticity peaks at 222 nm and 207 nm, corresponding to α-helices and β-sheets, respectively. However, upon incubation with each compound at its IC_50_ concentration, a decrease in these characteristic signals was observed, indicating conformational disruption. The most pronounced loss of secondary structure occurred in the presence of BZM-7, followed by CNZ-3, while BZM-9 showed minimal alterations compared to the native enzyme. The spectra of BZM-7 and CNZ-3 approached the profile of the buffer-only control, suggesting partial protein unfolding. These findings suggest that the loss of G6PD catalytic activity may be associated with the disruption of its native conformation.

Interestingly, the destabilization of the G6PD secondary structure by BZM-7 was comparable to that previously reported for JMM-2, which caused an ~80% loss of α-helical content in recombinant human G6PD [76]. These results indicate that all compounds affect the secondary structure of G6PD, which explains the loss of catalytic activity.

#### 2.5.3. Intrinsic Fluorescence Assays

**Figure 8:**
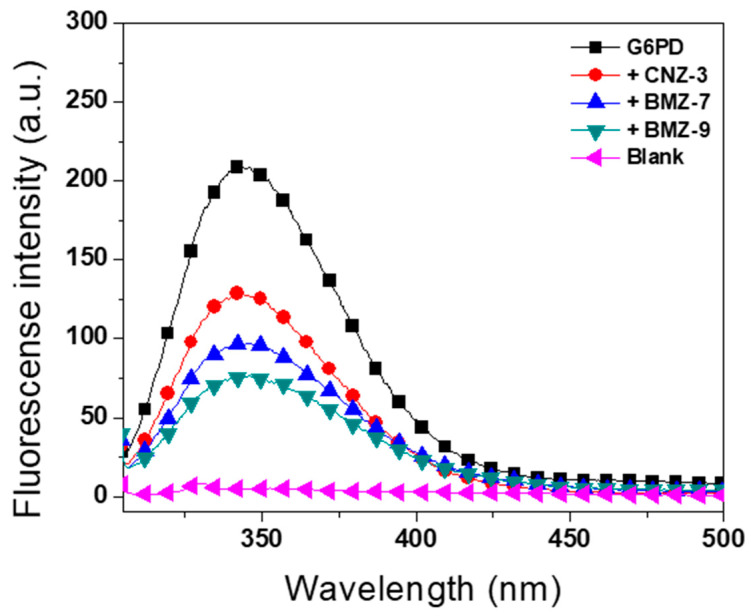
Intrinsic fluorescence spectra of recombinant human Glucose-6-Phosphate Dehydrogenase (G6PD) in the absence and presence of synthetic compounds. The enzyme was adjusted to a concentration of 0.1 mg/mL and incubated with the IC_50_ concentrations of each compound for 2 h at 37 °C. Spectra are representative of three independent experiments.

To further assess compound-induced structural perturbations at the tertiary level, intrinsic fluorescence spectroscopy was performed. The fluorescence signal of G6PD arises primarily from its seven tryptophan residues per monomer, which are sensitive to environmental changes in protein folding. As shown in Figure 8, a general decrease in intrinsic fluorescence was observed in the presence of compounds compared to the native enzyme without compounds. The compound-free enzyme exhibited a fluorescence intensity of 209 arbitrary units (a.u.), while in the presence of BZM-7, BZM-9, and CNZ-3, fluorescence values decreased to 97, 75, and 127 a.u., respectively. BZM-9 caused the greatest reduction (65%), followed by BZM-7 (54%), whereas CNZ-3 showed the smallest effect, with a 40% decrease.

These results suggest that BZM-7 and BZM-9, both benzimidazole-based compounds, induce marked conformational changes in G6PD’s tertiary structure, contributing to enzymatic inactivation. Although CNZ-3 exhibited a milder effect, it still altered the protein’s folding environment.

Similar structural disruptions were previously reported for JMM-2, which reduced G6PD fluorescence by ~50% [76] further supporting the idea that G6PD inhibition by small molecules involves destabilization of both secondary and tertiary structures.

#### 2.5.4. Molecular Docking of G6PD and Binding Site Prediction of BZM-7, BZM-9 and CNZ-3 Compounds on G6PD

**Figure 9:**
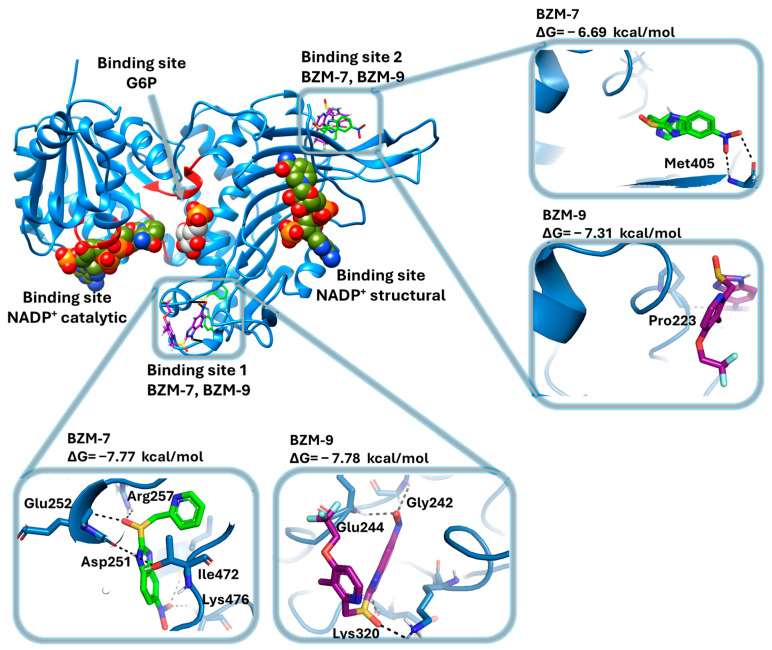
Representation of the model of the recombinant human Glucose-6-Phosphate Dehydrogenase (G6PD) monomer. The model highlights the binding sites for G6P, NADPH, and the benzimidazole compounds (BZM-7 and BZM-9). Binding site 1 (lower panel) and binding site 2 (upper panel) of BZM-7 and BZM-9 in the binding site. BZM-7 and BZM-9 are shown in green and purple, respectively; hydrogen bonds are represented by black dashed lines. The figure was generated using PyMOL Molecular Graphics System, version 2.5.0.

After performing in vitro cytotoxicity assays on glioblastoma cell lines and identifying the G6PD enzyme as a potential target of the nitrobenzimidazole compounds, molecular docking simulations were conducted to explore the possible molecular mechanisms of action of BZM-7 and BZM-9. Additionally, the binding modes of the nitrobenzimidazole compounds were compared with the nitrothiazole CNZ-3, whose molecular docking had already been performed [76]. To achieve this objective, we performed molecular docking studies using the G6PD monomer from *H. sapiens* (G6PD; PDB ID: 2BH9). Figure 9 shows the results of molecular docking analysis, which reveals that both BZM-7 and BZM-9 bind to two distinct non-catalytic regions of the G6PD surface. Neither compound docked within the catalytic site, suggesting a non-competitive inhibition mechanism, although this hypothesis requires further validation through kinetic studies.

For BZM-7, two major binding regions were identified (Figure 9). On binding site 1, 35% of the docked conformers clustered, and the most stable pose exhibited a binding free energy (ΔG) of −7.77 kcal/mol. This conformation formed two hydrogen bonds between the nitro group and residues Ile472 and Lys476, as well as an additional hydrogen bond between the sulfoxide group and Arg257. On binding site 2 (Figure 9), which is located near the structural NADP^+^ binding site and a β-sheet close to the dimerization interface, 45% of the conformers were found. The most stable pose in this site displayed a ΔG of −6.69 kcal/mol and formed two hydrogen bonds between the nitro group and Met405.

For BZM-9, the docking analysis identified the same two main binding regions as for BZM-7. On binding site 1 (Figure 9), 45% of the conformers clustered, and the most stable conformer showed a ΔG of −7.78 kcal/mol, forming two hydrogen bonds between the nitro group and residue Gly242, one between the sulfoxide group and Lys320, and another between the benzimidazole ring and Asp258. Binding site 2, included 51% of conformers, with a ΔG of −7.31 kcal/mol and one hydrogen bond between the nitro group and Pro223.

Importantly, binding site 1 is located near Asp258, a residue whose side chain has been described to interact with two hydroxyl groups of the glucose-6-phosphate (G6P) substrate, facilitating its proper positioning [82]. These interactions are conserved in the G6PD from *Leuconostoc mesenteroides* [83], suggesting that the binding of BZM-7 and BZM-9 to this region may interfere with substrate recognition. Additionally, two class I G6PD variants, Zacatecas and Waine, involve mutations at Arg257 (substituted by Leu and Gly, respectively). Biochemical characterization of the Zacatecas variant showed that the Arg257Leu substitution reduces G6P affinity by 35% and NADP^+^ affinity by 26% [84], emphasizing the functional relevance of this region. The site located near the structural NADP^+^ binding site is also critical for enzyme activity. A known Thr402Asn mutation results in the class I Covão do Lobo variant, which exhibits severely impaired catalytic activity in erythrocytes [85]. Thus, binding BZM-7 and BZM-9 to this region likely disrupts enzyme function.

**Figure 10:**
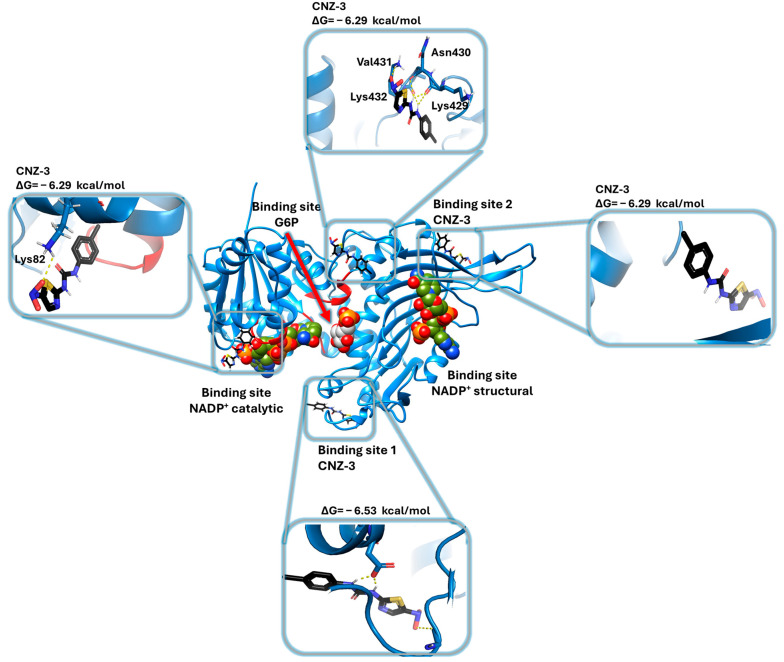
Representation of the model of the recombinant human Glucose-6-Phosphate Dehydrogenase (G6PD) monomer. The model highlights the binding sites for G6P, NADPH, and the nitrothiazole compound (CNZ-3). CNZ-3 is shown in black; hydrogen bonds are represented by black dashed lines. The figure was generated using PyMOL Molecular Graphics System, version 2.5.0.

These in silico findings suggest that the observed inhibition of G6PD activity by these compounds may be attributed to their interactions with structurally and functionally essential regions of the enzyme. The predicted non-catalytic binding supports a potential allosteric or non-competitive mechanism of action, which may underlie the potent antiproliferative activity of these compounds in glioblastoma cells. When comparing the binding sites found for nitrobenzimidazoles with the compound CNZ-3, it is observed that nitrothiazole also binds to the two sites found for BZM-7 and BZM-9, however CNZ-3 additionally shows affinity for two other zones, one of them is a pocket localized very close to the structural NADP^+^ binding site and the other binding site corresponds to an area very close to the active site of G6PD (Figure 10) [76]. These results are consistent with the previously observed non-competitive inhibition of the G6P substrate, such that CNZ-3 could be affecting the correct binding of the G6PD substrate and, consequently, decreasing its catalytic activity.

While the findings of the present study provide a promising indication of the antiproliferative activity of the compounds BZM-7, BZM-9 and CNZ-3, there are some important limitations to consider. First, experiments were performed only on ATCC glioblastoma cell lines. Although these models are well characterized and widely used in glioblastoma research, they do not replicate the complex three-dimensional tumor microenvironment, including interactions with blood vessels, immune cells, and stroma, which are crucial for tumor growth and therapeutic response. Furthermore, cytotoxicity assessment was limited to viability assays using MTT; however, this technique does not provide information on the type of cell death induced by the tested compounds. Considering that BZM-7 and BZM-9 are benzimidazole derivatives and CNZ-3 is a thiazole derivative, it is plausible that these compounds may activate distinct regulated cell death pathways, such as apoptosis, autophagy, or ferroptosis, as described for structurally related molecules [86,87,88,89,90]. Therefore, future studies should investigate these potential mechanisms using apoptosis/necrosis assays, caspase activity, and other biochemical markers. It is also advisable to extend the evaluations on physiologically relevant models, such as spheroids, organoids, clinical glioblastoma samples, or xenograft systems, to validate these initial results and strengthen translational applicability. In addition, although the observed reduction in VEGF expression is consistent with the proposed mechanism of action, this finding should be interpreted with caution. Further studies are required to clarify whether VEGF modulation by these compounds contributes directly to their cytotoxic activity or represents an indirect effect of G6PD inhibition.

## 3. Materials and Methods

### 3.1. Establishment of Monolayer Cell Cultures

The glioma cell lines U87-MG and A172, obtained commercially from the American Type Culture Collection (ATCC) (Manassas, VA, USA), were used in this study, with the HMC3 human microglial cell line serving as a non-tumoral control. The U87-MG and A172 glioblastoma lines were kindly provided by Dr. Benjamín Pineda Olvera from the National Institute of Neurology and Neurosurgery “Manuel Velasco Suárez,” while the HMC3 control line was donated by Dr. Beatriz Hernández Ochoa from the “Federico Gómez” Children’s Hospital of Mexico. Cells were cultured under normoxic conditions following ATCC recommendations (37 °C, 5% CO_2_ atmosphere), and hypoxic conditions were simulated using a 1% oxygen atmosphere. In general, cultures were incubated for 48 h in either EMEM (Eagle’s Minimum Essential Medium) or DMEM (Dulbecco’s Modified Eagle’s Medium), both supplemented with 10% Fetal Bovine Serum (FBS; Gibco, Waltham, MA, USA), 100 mg/mL penicillin, and 100 mg/mL streptomycin.

### 3.2. Screening of Chemical Compounds (On Cell Viability)

To assess the effects of the compounds on the viability of U87-MG and A172 glioblastoma cell lines, an initial screening assay was performed under normoxic conditions. A panel of nine chemical compounds was evaluated. As controls, the commercial inhibitor 6-aminonicotinamide (6AN) and acetylsalicylic acid (ASS)—previously reported as a G6PD inhibitor [91] were included. Additionally, seven synthetic benzimidazole-derived compounds (BZM-1, BZM-2, BZM-3, BZM-6, BZM-7, BZM-8, and BZM-9) [56], as well as JMM-2 [76] and CNZ-3 [92] were tested.

Once cultures reached confluence, cells were detached using a 0.5% trypsin solution and centrifuged at 1500× *g* in an Eppendorf 5810R^®^ (Hamburg, Germany) centrifuge for 3 min. The cells were then seeded into 96-well plates at a density of 1.0 × 10^4^ cells in 100 µL of culture medium per well. Plates were incubated for 24 h under the previously described conditions. Subsequently, cells were exposed to a fixed concentration of 250 µM of each compound. After 48 h, the medium was removed and 100 μL MTT (1 mg/mL in DMEM medium) was added to each well and incubated for 4 h at 37 °C. Then the medium was removed, and acid isopropanol was added to dissolve the blue formazan salts. Absorbance was recorded at 570 nm using a Multiskan Go microplate reader (Thermo Scientific, Waltham, MA, USA), and the data were analyzed using GraphPad Prism software version 8.0.2 considering the cells without a compound as 100% of MTT viability.

### 3.3. Determination of IC_50_ of Selected Compounds

Compounds that reduced cell viability by more than 50% at the fixed concentration of 250 µM in both glioblastoma cell lines were selected for further evaluation. BZM-7, BZM-9, and CNZ-3 met this criterion. To determine which compound had the greatest effect on cell viability, the half-maximal inhibitory concentration (IC_50_) was calculated.

Both cell lines were cultured to confluence. The cells were then seeded into 96-well plates at a density of 1.0 × 10^4^ cells in 100 µL of culture medium per well. Plates were incubated for 24 h under the previously described conditions. Subsequently, cells were treated with increasing concentrations (0 to 250 µM) of each compound (BZM-7, BZM-9, and CNZ-3). After 48 h of incubation, cell viability was measured using the MTT assay, as previously described. The dose–response curves were plotted by using GraphPad Prism software. The 50% cytotoxicity concentration (CC_50_) was calculated.

### 3.4. Evaluation of the Effect of Compounds on the Expression Levels of Glioblastoma Cell Lines Genes Using Quantitative RT-qPCR

To evaluate the metabolic and transcriptional effects of the compounds, the expression levels of genes involved in G6PD regulation (*SIRT2* and *KAT9*) and *VEGF* signaling were analyzed in A172 and U87-MG glioblastoma cell lines. Cells were cultured according to the previously described conditions. Primer sequences were obtained from GenBank Table 2.

Treatments were carried out using 1 × 10^4^ cells, incubated for 48 h at 37 °C under normoxic and hypoxic conditions, in the presence of each compound’s IC_50_ concentration. Total RNA was extracted using the TRIzol^®^ reagent (Thermo Fisher Scientific, Waltham, MA, USA) according to the manufacturer’s protocol. RNA concentration and purity were assessed using a NanoDrop^®^ ND-1000 spectrophotometer (NanoDrop Technologies, Wilmington, DE, USA). All RNA samples had A260/280 ratios between 2.0 and 2.2. RNA integrity was confirmed by 0.8% (*w*/*v*) agarose gel electrophoresis.

Complementary DNA (cDNA) was synthesized using 1 µg of DNase-treated total RNA. DNase II (Thermo Scientific) was used to remove genomic DNA contamination. The reverse transcription reaction (final volume: 20 µL) included DNase-treated RNA, 10 mM dNTP mix, oligo(dT)_18_ primers, and RevertAid reverse transcriptase (Thermo Scientific). The mixture was incubated at 42 °C for 60 min, followed by enzyme inactivation at 70 °C for 10 min. The resulting cDNA was stored at −20 °C until further analysis. cDNA concentration and purity were again verified using the NanoDrop^®^ ND-1000.

For RT-qPCR analysis, specific primers were designed for genes by primer 3 software, including glucose-6-phosphate dehydrogenase (*G6PD*), sirtuin 2 (*SIRT2*), elongator acetyltransferase complex subunit 3 (*KAT9*), and vascular endothelial growth factor A (*VEGF*). Primer design was based on NCBI database sequences, following standard parameters: primer length 18–20 bp, melting temperature (Tm) ~ 61 °C, GC content ~ 40%, and amplicon size between 80 and 200 bp (Table 1). Oligonucleotides were synthesized by the Sequencing and Synthesis Unit at the Institute of Biotechnology, UNAM.

Amplification was performed using a StepOne™ Real-Time PCR System and the Fast SYBR^®^ Green Master Mix kit (Applied Biosystems, Foster City, CA, USA) under the following conditions: initial denaturation at 95 °C for 30 s, followed by 40 cycles of 95 °C for 3 s and 60 °C for 30 s. A melt curve analysis was conducted after amplification by gradually increasing the temperature from 60 °C to 95 °C.

To quantify expression levels, 100 ng of cDNA from the control HMC3 cell line, as well as from untreated and compound-treated A172 and U87-MG glioblastoma cell cultures (BZM-7, BZM-9, and CNZ-3), was used. *Pyruvate kinase* (*PKM*) was employed as the reference gene [56]. Normalization was performed using the geometric mean of Ct values, and relative gene expression was calculated using the 2^−ΔΔCt^ method [93]. Each reaction was run in quintuplicate for all analyzed genes and cell lines. Results are presented as mean values ± standard deviation (SD).

### 3.5. Heterologous Expression of Human Glucose-6-Phosphate Dehydrogenase (G6PD)

Biochemical and physicochemical assays were carried out using recombinant human G6PD. For overexpression, *Escherichia coli* BL21(DE3)Δzwf::kan^r^ competent cells were transformed with the pET3a expression vector carrying the human *G6PD* gene (accession no. NM_001042351.2) [94]. The endogenous *zwf* gene, which encodes native G6PD in *E. coli*, was disrupted and replaced by a kanamycin resistance cassette to allow exclusive expression of the recombinant human enzyme.

The *E. coli* BL21(DE3)Δzwf::kan^r^ strain containing the pET3a/*G6PD* plasmid was used to inoculate 2 L of Luria–Bertani medium supplemented with 100 µg/mL ampicillin. Cultures were incubated at 37 °C with constant agitation at 180 rpm. Recombinant protein expression was induced by adding 0.3 mM isopropyl-β-D-1-thiogalactopyranoside (IPTG) when the culture reached an optical density of 0.8 at 600 nm, followed by incubation at 25 °C for 18 h under the same agitation conditions [95]. After incubation, cells were harvested by centrifugation at 5000× *g* for 20 min using an Eppendorf 5810R^®^ centrifuge. The resulting cell pellet was then used for protein purification.

### 3.6. Purification of Recombinant Human Glucose-6-Phosphate Dehydrogenase (G6PD)

To evaluate the effects of synthetic compounds on the catalytic and physicochemical properties of G6PD, the recombinant enzyme was purified. Bacterial pellets were resuspended in 30 mL of lysis buffer (50 mM K_2_HPO_4_, 0.1% β-mercaptoethanol, and 0.5 mM PMSF in 0.1% DMSO). Cell disruption was performed as previously described [94,95]. The lysate was centrifuged at 9000× *g* for 30 min, and the supernatant was collected as the crude enzyme extract.

Purification of G6PD was performed according to the protocol described by [94] employing 2′,5′-ADP Sepharose 4B affinity chromatography and Q-Sepharose 4B anion-exchange chromatography (Sigma-Aldrich, St. Louis, MO, USA). The crude extract was first loaded onto a Q-Sepharose 4B column, which was equilibrated with 50 mM K_2_HPO_4_ buffer (pH 7.35). The column was washed with five column volumes (50 mL) until the absorbance at 280 nm returned to baseline. Elution was carried out using a linear NaCl gradient from 0 to 350 mM in the same buffer. Each 2 mL fraction was assayed for G6PD activity using a standard reaction mixture (100 mM Tris-HCl, 3 mM MgCl_2_, 1 mM G6P, and 1 mM NADP^+^, at pH 8.0). Fractions showing enzymatic activity were concentrated using 30 kDa Centricon concentrators (Millipore^®^, Billerica, MA, USA), and the resulting concentrate was applied to a 2′,5′-ADP Sepharose 4B affinity column (GE Healthcare^®^, Piscataway, NJ, USA) equilibrated with the same buffer. The column was washed with five column volumes until the absorbance at 280 nm was close to zero. Protein elution was achieved using the equilibration buffer supplemented with 100 µM NADP^+^. Eluted fractions were assayed for enzymatic activity by monitoring NADPH production at 340 nm using the standard reaction mixture. Fractions with G6PD activity were further concentrated using 30 kDa Centricon units (Millipore^®^).

The purity of the G6PD enzyme was assessed by 12% SDS-PAGE followed by staining with colloidal Coomassie Brilliant Blue R-250 (Sigma-Aldrich). Protein concentration was determined using the modified Lowry assay [96], with bovine serum albumin as the standard. The purified enzyme was immediately used for the experimental assays described in this study.

### 3.7. Selection of Glucose-6-Phosphate Dehydrogenase (G6PD) Inhibitors

Compounds that have previously been shown to reduce cell viability were selected for further analysis. Three chemical compounds were evaluated to identify potential inhibitors of G6PD catalytic activity. Each compound was dissolved in DMSO at a final concentration of 5%, resulting in a final concentration of 250 µM. The compounds were incubated with 0.2 mg/mL of purified recombinant G6PD at 37 °C for 2 h. Following incubation, the residual enzymatic activity of G6PD was measured using a standard reaction mixture, initiating the enzymatic reaction with 200 ng of the incubated protein. To confirm that 5% DMSO did not interfere with enzymatic activity, a control was included in which the protein was incubated with DMSO alone. It was verified that this concentration of DMSO did not affect G6PD activity, and the activity under this condition was considered as 0% inhibition. Residual G6PD activities for each compound were normalized against this control.

### 3.8. Concentration–Response Inactivation Assay and Determination of IC_50_ Values

The selected compounds were used to determine the half-maximal inhibitory concentration (IC_50_), which represents the concentration required to reduce the enzyme’s activity by 50%. Purified G6PD enzyme (0.2 mg/mL) was incubated with increasing concentrations (0–400 µM) of each selected compound for 2 h at 37 °C. After incubation, residual enzyme activity was measured using a standard reaction mixture by monitoring NADPH production at 340 nm. Initial reaction rates were normalized to the activity of the enzyme without inhibitor, which was set as 100% residual activity (Control). Residual activities were plotted as percentages on the y-axis, and inhibitor concentrations on the x-axis. All data were fitted to a Boltzmann sigmoidal equation using Origin 8.0^®^ software to calculate the exact IC_50_ values, which were expressed in µM.

### 3.9. Analysis of Secondary Structure by Circular Dichroism

To investigate whether the decrease in G6PD catalytic activity caused by the selected compounds was associated with alterations in the enzyme’s secondary structure, circular dichroism (CD) analysis was performed in the presence of these compounds. The assay was carried out using a Jasco J-810 spectropolarimeter (Jasco Inc., Easton, MD, USA) under a continuous flow of high-purity nitrogen. The protein was prepared at a concentration of 0.2 mg/mL in 50 mM phosphate buffer, pH 7.35, and incubated with the IC_50_ concentration of each inhibitor for 2 h at 37 °C. The secondary structure was evaluated in the far-UV region (200–240 nm), focusing on changes in molar ellipticity at 222 and 208 nm, which are characteristic of α-helices and β-sheets, respectively. As a control, phosphate buffer mixed with each inhibitor was used, and their spectra were subtracted from the spectra of the samples containing both the protein and the inhibitors, following the protocol described by [95].

### 3.10. Structural Analysis by Intrinsic Fluorescence

To evaluate whether the decrease in G6PD activity caused by the inhibitors is associated with alterations in its tertiary structure, intrinsic fluorescence assays were conducted in the presence of each inhibitor at their respective IC_50_ concentrations. These experiments were carried out using a PerkinElmer LS-55 spectrofluorometer (Perkin Elmer, Wellesley, MA, USA), with the G6PD protein prepared at a concentration of 0.1 mg/mL in 50 mM phosphate buffer, pH 7.35. The protein was incubated for 2 h at 37 °C with each inhibitor. Intrinsic fluorescence was measured by exciting the samples at 295 nm, and emission spectra were recorded from 310 to 500 nm, using 10 nm slit widths for both excitation and emission. All assays were performed in triplicate to ensure the reproducibility of the results [97].

### 3.11. Molecular Docking Studies

#### 3.11.1. Structure of the G6PD Protein and Ligands

To predict the binding interactions of compounds BZM-7 and BZM-9 with the G6PD enzyme, blind molecular docking analyses were performed using the crystal structure of human G6PD available in the Protein Data Bank (PDB ID: 2BH9). The structure was pre-processed, evaluated, and validated, with hydrogen atoms added using the MolProbity online server (http://molprobity.biochem.duke.edu/, accessed on 1 August 2025).

#### 3.11.2. Blind Molecular Docking Study

The molecular docking analysis was performed on the entire surface of the G6PD protein with the benzimidazole chemicals. Docking was performed using the SwissDock online server (http://old.swissdock.ch/docking, accessed on 1 August 2025) to predict the molecular interactions that may occur between the G6PD protein and the compounds BZM7 and BZM9. The most stable ligand poses were selected based on the following criteria: (1) clustering of ligand conformers on the protein surface, (2) free binding energy (ΔG) of the protein–inhibitor complex, and (3) the type of chemical interactions formed (e.g., hydrogen bonds or covalent interactions). These analyses allowed the prediction of potential binding sites on the G6PD protein. The conformers with the lowest free energy values and strongest predicted interactions were considered to be the most likely to exert high inhibitory activity against G6PD.

## 4. Conclusions

Overall, the findings of this study demonstrate the cytotoxic potential of the nitrobenzimidazole derivatives BZM-7 and BZM-9, as well as the nitrothiazole compound CNZ-3, in glioblastoma cell lines under both normoxic and hypoxic conditions. Notably, all three compounds exhibited antiproliferative activity in U87-MG and A172 glioblastoma cell lines, particularly under hypoxia. Regarding its possible mechanism of action, it was found that the G6PD enzyme was identified as a potential pharmacological target. In vitro enzymatic assays revealed that BZM-7, BZM-9, and CNZ-3 inhibit G6PD activity at lower concentrations than previously reported inhibitors. Molecular docking analyses further predicted that these compounds bind to key non-catalytic but functionally relevant regions of the enzyme, likely inducing conformational changes that disrupt its catalytic function. At the transcriptional level, treatment with the nitrocompounds resulted in the downregulation of *G6PD*, *SIRT2*, and *VEGF* gene expression, which may contribute to reduced glioblastoma cell proliferation and impaired adaptation to hypoxic stress. It is well established that drugs containing nitroaromatic groups can induce idiosyncratic toxicity, which is a major reason they are often avoided in drug design and considered structural liabilities. Nonetheless, the nitro group can also function as both a pharmacophore and a selective toxicophore [98]. Moreover, nitrobenzimidazole and nitrothiazole derivatives have been identified as ‘toxicity cliffs’ due to their potent and highly selective cytotoxic activity [99]. Taken together, these results suggest that nitrocompounds BZM-7, BZM-9, and CNZ-3 are promising candidates for further investigation as potential therapeutic agents for glioblastoma, particularly by targeting redox homeostasis and metabolic adaptation pathways.

## Figures and Tables

**Table 1:** IC_50_ of BZM-7, BZM-9 and CNZ-3 in A172 and U87-MG glioblastoma cells lines under normoxic and hypoxic conditions.

	A172			U87-MG		
	BZM-7	BZM-9	CNZ-3	BZM-7	BZM-9	CNZ-3
Normoxia	43 ± 1.6 µM	35 ± 1.6 µM	12 ± 1.0 µM	38 ± 1.6 µM	48 ± 1.6 µM	12 ± 1.3 µM
Hypoxia	43 ± 1.6 µM	125 ± 2.6 µM	9 ± 0.9 µM	33 ± 1.5 µM	44 ± 1.6 µM	19 ± 1.4 µM

**Table 2:** Primers designed for evaluating expression levels using RT-qPCR.

Gene	5′-3′ Sequence	Length (bp)	Function
PKM, *pyruvate kinase M1/2*, *variant 1*	Fw 5′-GGTTCGGAGGTTTGATGA-3′ Rv 5′-GGCTTCTTGATCATGCTCT-3′	186	Glycolysis
G6PD, *glucose-6-phosphate dehydrogenase variant 1*	Fw 5′-ATATTTATGGCAGCCGAGG-3′ Rv 5′-GTCAATGGTCCCGGTGT-3′	190	Pentose phosphate pathway (PPP)
SIRT2, *sirtuin 2*	Fw 5′-TTGGATGGAAGAAGGAGC-3′ Rv 5′-AGCTGTCACTGGGGTTTCT-3′	153	Deacetylase involved in metabolism and stress response
ELP3, *elongator acetyltransferase complex subunit 3* *	Fw 5′-TGCTAGTGGGATTGCTGT-3′ Rv 5′-TCAGAATCAGGTCCACCA-3′	90	Lysine acetyltransferase, coactivator of transcription
VEGFA, *vascular endothelial growth factor A*	Fw 5′-TCTCTACCCCAGGTCAGACG-3′ Rv 5′-AGCAATGTCCTGAAGCTCCC-3′	98	Angiogenesis and hypoxia response

* The KAT9 gene is also referred to as ELP3 in NCBI GenBank (transcript NM_ NM_001284222.2), and primers were designed based on this reference sequence.

## Data Availability

Data are contained within the article.

## Abbreviations

The following abbreviations are used in this manuscript:

PPP	Pentose Phosphate Pathway
G6PD	Glucose-6-Phosphate Dehydrogenase
GBM	Glioblastoma
TMZ	Temozolomide
HIF-1α	Hypoxia-Inducible Factor 1-Alpha
VEGF	Vascular Endothelial Growth Factor
GSCs	Glioma Stem-Like Cells
PTMs	Post-Translational Modifications
KAT9	Lysine Acetyltransferase
6AN	6-aminonicotinamide
ASA	Acetylsalicylic Acid
IC_50_	Half-Maximal Inhibitory Concentration
CD	Circular Dichroism
G6P	Glucose-6-Phosphate
ATCC	American Type Culture Collection
EMEM	Eagle’s Minimum Essential Medium
DMEM	Dulbecco’s Modified Eagle’s Medium
FBS	Fetal Bovine Serum
MTT	3-(4,5-dimethylthiazol-2-yl)-2,5-diphenyltetrazolium bromide
cDNA	Complementary DNA
SIRT2	Sirtuin 2
SD	Standard Deviation
IPTG	isopropyl-β-D-1-thiogalactopyranoside
